# Impact of pneumococcal conjugate vaccines introduction on antibiotic resistance of *Streptococcus pneumoniae* meningitis in children aged 5 years or younger, Israel, 2004 to 2016

**DOI:** 10.2807/1560-7917.ES.2018.23.47.1800081

**Published:** 2018-11-22

**Authors:** Shalom Ben-Shimol, Noga Givon-Lavi, David Greenberg, Michal Stein, Orli Megged, Avihu Bar-Yochai, Shahar Negari, Ron Dagan, on behalf of the Israel Bacteremia and Meningitis Active Surveillance Group

**Affiliations:** 1 The Pediatric Infectious Disease Unit, Soroka University Medical Center, Beer-Sheva, Israel; 2 The Faculty of Health Sciences, Ben-Gurion University of the Negev, Beer-Sheva, Israel; 3 Infectious Diseases and Infection Control Unit, Hillel Yaffe Medical Center, Hadera, Israel and Rappaport Faculty of Medicine, Technion – Israel Institute of Technology, Haifa, Israel; 4 Pediatric Infectious Diseases Unit, Shaare Zedek Medical Center, affiliated with Hebrew University-Hadassah School of Medicine, Jerusalem, Israel; 5 Infectious Disease Unit, Assaf Harofe Medical Center, Zerifin, Israel; 6 Members of the Israel Bacteraemia and Meningitis Active Surveillance Group have been acknowledged at the end of the article

**Keywords:** Streptococcus pneumoniae, meningitis, antibiotic resistance, children, pneumococcal conjugate vaccines

## Abstract

**Background:**

Empiric treatment of pneumococcal meningitis includes ceftriaxone with vancomycin to overcome ceftriaxone resistant disease. The addition of vancomycin bears a risk of adverse events, including increased antibiotic resistance. We assessed antibiotic resistance rates in pneumococcal meningitis before and after pneumococcal conjugate vaccine (PCV) implementation.

**Methods:**

All pneumococcal meningitis episodes in children aged 5 years and younger, from 2004 to 2016, were extracted from the nationwide bacteremia and meningitis surveillance database. For comparison purposes, we defined pre-PCV period as 2004–2008 and PCV13 period as 2014–2016. Minimal inhibitory concentration (MIC) > 0.06 and > 0.5 μg/mL were defined as penicillin and ceftriaxone resistance, respectively.

**Results:**

Overall, 325 episodes were identified. Pneumococcal meningitis incidence rates declined non-significantly by 17%, comparing PCV13 and pre-PCV periods. Throughout the study, 90% of isolates were tested for antibiotic susceptibility, with 26.6%, 2.1% and 0% of isolates resistant to penicillin, ceftriaxone and vancomycin, respectively. Mean proportions (± SD) of meningitis caused by penicillin-resistant pneumococci were 40.5% ± 8.0% and 9.6% ± 7.4% in the pre-PCV and the PCV13 periods, respectively, resulting in an overall 83.9% reduction (odd ratio:0.161; 95% confidence interval: 0.059–0.441) in penicillin resistance rates. The proportions of meningitis caused by ceftriaxone resistant pneumococci were 5.0% ± 0.8% in the pre-PCV period, but no ceftriaxone resistant isolates were identified since 2010.

**Conclusions:**

PCV7/PCV13 sequential introduction resulted in > 80% reduction of penicillin- resistant pneumococcal meningitis and complete disappearance of ceftriaxone resistant disease. These trends should be considered by the treating physician when choosing an empiric treatment for pneumococcal meningitis.

## Introduction

Bacterial meningitis is a major cause of morbidity and mortality in children worldwide, with *Streptococcus pneumoniae* being the leading cause of bacterial meningitis in up to 60% of cases [1,2]. Pneumococcal meningitis has high rates of long-term complications (e.g. behavioural/intellectual disorders, hearing loss and neurologic deficits), with mortality rates of up to 26% [2].

The early and rapid administration of antibiotics is crucial to increase survival and reduce morbidity in pneumococcal meningitis and the choice of empiric antibiotics should be based on the local epidemiology of antibiotic susceptibility, among other factors [3]. Antibiotic resistance definitions for pneumococcal meningitis are stricter than those for non-meningitis pneumococcal infections, with minimal inhibitory concentrations (MIC) cut-offs of > 0.06 μg/mL for penicillin resistance and MIC > 0.5 μg/mL for ceftriaxone resistance, according to the Clinical and Laboratory Standards Institute (CLSI) [2,4].

Before the introduction of pneumococcal conjugate vaccines (PCVs) worldwide, an increase in pneumococcal antimicrobial (mainly penicillin) resistance had been observed [5-8], resulting in modifications of the empiric management of meningitis [8,9]. Initially, third generation cephalosporins (cefotaxime and ceftriaxone) became the standard therapy in children and subsequently, in the mid-1990s, reports of cephalosporin-resistant pneumococcal meningitis led to the recommendation of adding vancomycin as empiric therapy in suspected cases [3,10]. Therefore, the empiric treatment in cases of suspected pneumococcal meningitis currently includes intra-venous ceftriaxone and vancomycin pending culture results [10-12].

Due to its low penetration through the meninges, vancomycin is given at high doses (60mg/kg/day). The addition of empiric treatment with vancomycin may rarely result in side effects, such as hypersensitivity, nephrotoxicity (especially at high doses used to treat pneumococcal meningitis) and hearing loss [2,13]. Furthermore, using vancomycin increases the selection and exposure of bacteria to antibiotics and may accelerate the emergence of strains resistant to vancomycin, which is one of the antibiotics saved as last-resort treatment against bacteria resistant to other antimicrobial drugs [2,13-15].

In July 2009, the 7-valent pneumococcal conjugate vaccine (PCV7) was introduced to the Israeli national immunisation programme (NIP) and in November 2010, was replaced by the 13-valent pneumococcal conjugate vaccine (PCV13) [16]. By December 2012, the proportion of children who received two or more doses of PCV7 and PCV13 was greater than 95% [16]. Following PCV7/PCV13 sequential introduction, rates of pneumococcal meningitis and other invasive pneumococcal disease (IPD) caused by vaccine-serotype pneumococci substantially declined by ca 95% in children aged  5 years or younger [17]. In contrast, overall pneumococcal meningitis rates were reduced non-significantly by 27% and remaining cases mainly reflected an increased rate of non-vaccine serotype pneumococcal meningitis [17]. Besides the impact observed worldwide on IPD rates caused by vaccine-serotypes, including meningitis, PCV introduction in the United States (US) was also followed by a reduction in antibiotic resistance rate [18,19]. This reduction may have derived from the elimination of resistant vaccine strains and the increase in non-vaccine strains [17,18,20] that are more frequently antibiotic susceptible, as they were previously less exposed to antibiotic selection stress [18].

The empiric treatment of suspected pneumococcal meningitis with ceftriaxone and vancomycin both in Israel and in many settings worldwide [2,11] was rarely revisited following PCVs introduction. Nevertheless, vancomycin treatment might not be justified, if ceftriaxone resistance rates are shown to be low [3].

We assessed antibiotic resistance rates in pneumococcal meningitis episodes before and after PCV introduction to the Israeli NIP, in an attempt to optimise current treatment recommendations.

## Methods

The ongoing, prospective, nationwide, population-based and active surveillance study, conducted by the Israeli Paediatric Bacteraemia and Meningitis Group, was initiated in 1989. The current report describes data spanning over a 12-year period (July 2004–June 2016). Incidence rates of pneumococcal meningitis are presented from July 2000, to allow better appreciation of secular trends and fluctuations.

The study was approved by the Institutional Ethics Committees of the participating medical centres.

### Setting and study population

The study population comprised all children aged 5 years or younger in Israel. As of 2016, Israel had a population of ca 875,000 children in this age group [21].

The study has been conducted in all 26 medical health centres routinely obtaining cerebrospinal fluid (CSF) cultures from children [16,22], no CSF cultures are obtained outside these centres, enabling us to collect the majority of culture-confirmed pneumococcal meningitis cases in Israel.

Local investigators in each centre responded to a monthly distributed questionnaire sent by the principal investigator at the study headquarters.

### Case definitions

A pneumococcal meningitis episode was defined as an illness during which *S. pneumoniae* was isolated from either CSF or from blood with laboratory signs suggestive of meningitis (e.g. CSF pleocytosis). Non-culture diagnoses (polymerase chain reaction, antigen testing, Gram stain results or clinical diagnosis) were excluded. In Israel, < 5% of all meningitis episodes are diagnosed by non-culture methods.

### Antibiotic susceptibility testing

According to the National Committee for Clinical and Laboratory definitions for antibiotic susceptibility in pneumococcal meningitis, isolates with a MIC of > 0.06 μg/mL for penicillin were determined as penicillin resistant. Isolates with an MIC > 0.5 μg/mL for ceftriaxone were defined as ceftriaxone resistant [2,13].

### Uptake of pneumococcal conjugate vaccines

PCV7 NIP was initiated in July 2009 with a catch-up campaign in children aged 2 years or younger [17,23]. In November 2010, PCV13 replaced PCV7 without further catch-up.

Vaccine uptake evaluation methods were previously described [16,17]. By June 2011 and December 2012, ca 80% and ca 90%, respectively, of 7–11 month old children received two or more doses of PCV7 and/or PCV13 and ca 95% received two or more PCV13 doses by June 2014 and June 2015.

By June 2011 and December 2012, 36% and 87%, respectively, of children aged 24–35 months, received three or more PCV7/PCV13 doses and > 90% received three or more doses of PCV13 by June 2014 and June 2015.

### Data analysis

Annual (July to June) incidence rates were calculated as the number of CSF positive culture cases divided by the total population at risk during each year of the study [21].

For episodes in which the serotype and/or serogroup were missing, a detailed extrapolation was conducted, as described elsewhere [22]. Briefly, the proportion of episodes attributed to each serotype-group (vaccine type and non-vaccine type) was assigned from the age-and ethnicity-specific strata, assuming serotype data were missing at random.

Since 2009–2010, the proportion of isolates with serotype determination increased to > 95% from ca 60% in the pre-PCV period (2004–2008) [22].

To assess changes in meningitis incidence and proportions of antibiotic resistance (penicillin MIC > 0.06; ceftriaxone MIC > 0.5) of all isolates, we used annual rates. Data are presented for all study years (2004–2016). In addition, we compared rates in the pre-PCV period (July 2004–June 2008) and the last two study years (July 2014–June 2016) as the PCV13 period.

Incidences were calculated using the birth cohorts born in Israel, according to the Israeli Central Bureau of Statistics reports [21]. Incidence rate ratios (IRRs) and 95% confidence intervals (CIs) were calculated for meningitis rates.

Proportions of antibiotic resistance of all isolates and odds ratios (ORs) with 95% CI were calculated. P values of dynamics in proportions of antibiotic resistance were calculated.

Data were analysed with SPSS 23.00 software. Univariate analyses were conducted using two-tailed chi-squared test or Student t-test, where appropriate. A p value < 0.05 was considered statistically significant.

## Results

During the study period, 325 pneumococcal meningitis episodes were identified in children aged 5 years or younger. The mean age was 14.5 ± 13.7 months, and median age was 10.1 months, with 54.8% (n = 178) of children aged 12 months of less. Of the 325 episodes, 53.2% (n = 173) were in males.

Of all 325 isolates, 289 (88.9%) and 291 (89.5%) were tested for penicillin and ceftriaxone susceptibility, respectively. Overall, throughout the study, 77 (26.6%) isolates were penicillin-resistant and six (2.1%) isolates were ceftriaxone-resistant. All (n = 290) tested isolates were susceptible to vancomycin.

### Pneumococcal meningitis rates dynamics

**Figure 1:**
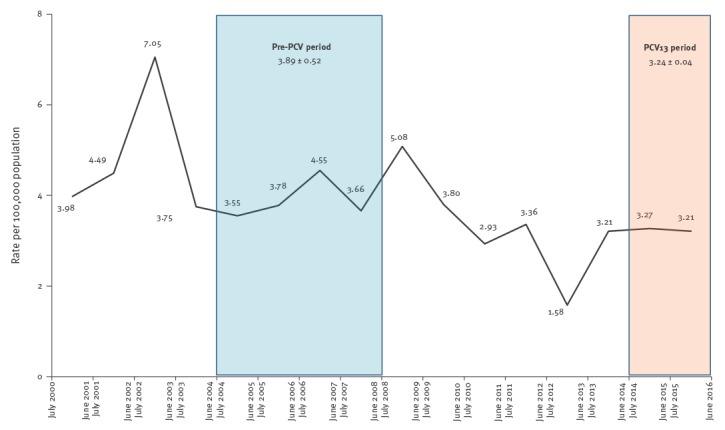
Pneumococcal meningitis incidence rates in children aged 5 years and younger, Israel, July 2000–June 2016 (n = 455)

Following PCV13 introduction, meningitis rates declined non-significantly by 17% (IRR: 0.83; 0.60–1.15) comparing the PCV13 and the pre-PCV periods (Table 1, Figure 1).

**Table 1:** Number of pneumococcal meningitis episodes and incidence rates in children aged 5 years or younger, Israel, July 2000–June 2016

Time period	Pneumococcal meningitis	
	Number of episodes	Incidence per 100,000
July 00–June 01	26	3.98
July 01–June 02	30	4.49
July 02–June 03	48	7.05
July 03–June 04	26	3.75
July 04–June 05^a^	25	3.55
July 05–June 06^a^	27	3.78
July 06–June 07^a^	33	4.55
July 07–June 08^a^	27	3.66
July 08–June 09	38	5.08
July 09–June 10	29	3.80
July 10–June 11	23	2.93
July 11–June 12	27	3.36
July 12–June 13	13	1.58
July 13–June 14	27	3.21
July 14–June 15^b^	28	3.27
July 15–June 16^b^	28	3.21
**IRR (95% CI)**		
PCV 13 vs pre-PCV	NA	0.83 (0.60–1.15)

CI: confidence interval; IRR: incidence rate ratio; NA: not available; PCV: pneumococcal conjugate vaccine.

^a^ Period termed as ‘Pre-PCV period’ defined as 2004–­2008.

^b^ Period termed as ‘PCV 13 period’ defined as 2014–­2016.

PCV: pneumococcal conjugate vaccine.

‘Pre-PCV period’ defined as 2004–2008 and ‘PCV 13 period’ defined as 2014–2016.

Incidence rates per 100,000 children.

### Proportions of penicillin-resistant isolates

**Figure 2:**
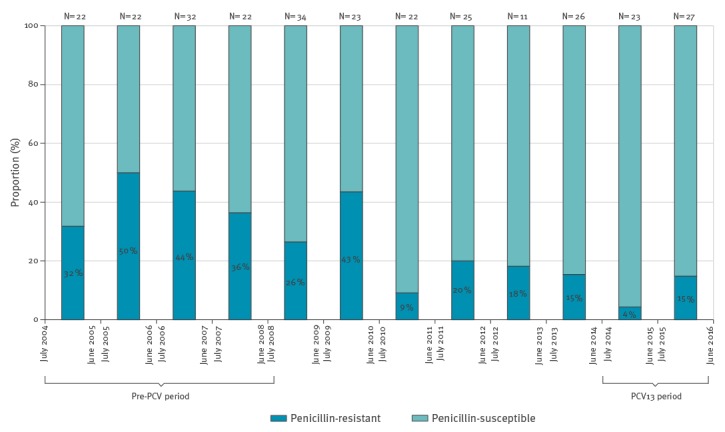
Proportions of penicillin-resistant pneumococcal meningitis of all isolates in children aged 5 years or younger, Israel, July 2004–June 2016 (n = 289)

In the pre-PCV period, proportions of meningitis caused by penicillin-resistant pneumococci were 40.5% ± 8.0% (Table 2, Figure 2). In the PCV13 period, these proportions significantly declined by 83.9% (OR = 0.16; 0.06–0.44) and were 9.6% ± 7.4%. Similarly, a significant declining trend of proportions of penicillin-resistant pneumococcal meningitis within pneumococcal isolates is presented in supplement.

**Table 2:** Proportions of penicillin-resistant and ceftriaxone-resistant pneumococcal meningitis of all isolates in children aged 5 years and younger, Israel, July 2004–June 2016

Time period	Penicillin MIC > 0.06 μg/mL		Ceftriaxone MIC > 0.5 μg/mL	
	Number of isolates	Percent	Number of isolates	Percent
July 04–June05^a^	7/22	31.8	1/22	4.5
July 05–June 06^a^	11/22	50.0	1/20	5.0
July 06–June 07^a^	14/32	43.8	2/33	6.1
July 07–June 08^a^	8/22	36.4	1/23	4.3
July 08–June 09	9/34	26.5	0/36	0.0
July 09–June 10	10/23	43.5	1/22	4.5
July 10–June 11	2/22	9.1	0/22	0.0
July 11–June 12	5/25	20.0	0/25	0.0
July 12–June 13	2/11	18.2	0/11	0.0
July 13–June 14	4/26	15.4	0/26	0.0
July 14–June 15^b^	1/23	4.3	0/23	0.0
July 15–June 16^b^	4/27	14.8	0/28	0.0
**OR (95% CI)**				
PCV 13 vs pre PCV	NA	**0.16 (0.06–0.44)**	NA	NA

CI: confidence interval; MIC: Minimal inhibitory concentration; NA: not available; OR: odds ratio; PCV: pneumococcal conjugate vaccine.

^a^ Period termed as ‘Pre-PCV period’ defined as 2004–2008.

^b^ Period termed as ‘PCV 13 period’ defined as 2014–2016.

PCV: pneumococcal conjugate vaccine.

Penicillin-resistant pneumococcal meningitis = minimum inhibitory concentration (MIC) > 0.06 μg/mL.

‘Pre-PCV period’ defined as 2004–2008 and ‘PCV 13 period’ defined as 2014–2016.

### Proportions of ceftriaxone-resistant pneumococcal isolates in pneumococcal meningitis

**Figure 3:**
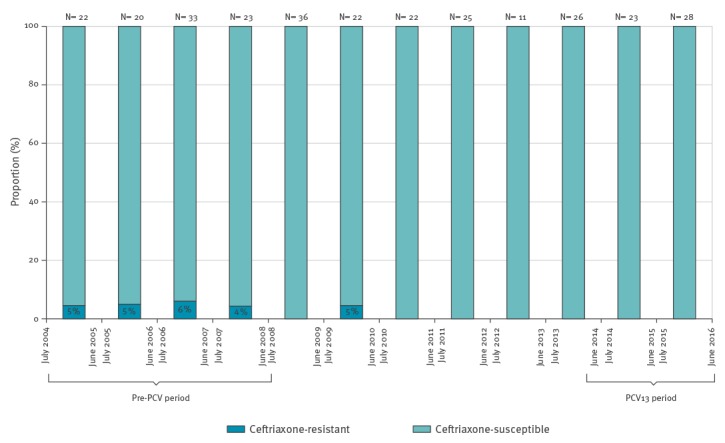
Proportions of ceftriaxone-resistant pneumococcal meningitis of all isolates in children aged 5 years and younger, Israel, July 2004–June 2016 (n = 291)

In the pre-PCV period, proportions of meningitis caused by ceftriaxone-resistant pneumococci were 5.0% ± 0.8% (Table 2, Figure 3). Following PCV13 introduction, these proportions declined to 0.0%. No cases of ceftriaxone resistant isolates were observed since July 2010.

PCV: pneumococcal conjugate vaccine.

Ceftriaxone-resistant pneumococcal meningitis = minimal inhibitory concentration > 0.5 μg/mL.

‘Pre-PCV period’ defined as 2004–2008 and ‘PCV 13’ period defined as 2014–2016.

### Proportions of PCV13 serotypes (VT13), and non-VT13 pneumococcal isolates in antibiotic-resistant meningitis

**Figure 4:**
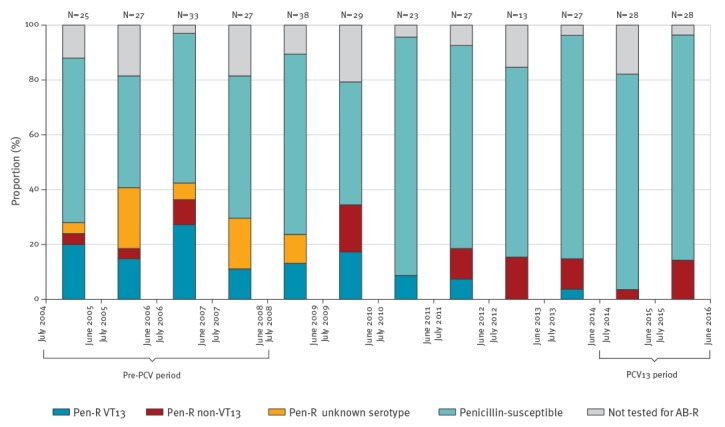
Proportions of PCV13 serotypes of all penicillin-resistant pneumococcal meningitis isolates in in children aged 5 years or younger, Israel, July 2004–June 2016 (n = 325)

Of 40 penicillin-resistant isolates in the pre-PCV period, 26 had a known serotype. Since July 2009, all 28 penicillin resistant isolates had known serotypes. Of 26 resistant isolates in the pre-PCV period, 21 were VT13, while in the PCV13 period, none (0/5) of the resistant isolates were VT13 (Figure 4).

PCV: pneumococcal conjugate vaccine.

‘Pre-PCV period’ defined as 2004–2008 and ‘PCV 13 period’ defined as 2014–2016.

Within the non-VT13 group, penicillin resistance rates declined from 33.3% in the pre-PCV period to 10.4% in the PCV13 period (p = 0.049).

For the six ceftriaxone resistant isolates (all before July 2010), four isolates had a known serotype; all three isolates from the pre-PCV period were VT13 and the one isolate from the year 2009–2010 was non-VT13.

## Discussion

The sequential introduction of PCV7/PCV13 into the Israel NIP resulted in an 80% decline in the incidence of antibiotic-resistant pneumococcal meningitis, even though the overall pneumococcal meningitis incidence rates did not decline significantly; reflecting the near elimination of disease caused by vaccine serotypes.

Before PCV implementation, vaccine-serotypes were the most successful serotypes in nasopharyngeal colonisation and therefore the most frequent pathogens causing pneumococcal diseases [24]. This frequent and relatively prolonged colonisation and involvement in disease resulted in continuous antibiotic pressure (selection for an antibiotic-resistant strain deriving from frequent or prolonged exposure to antibiotics) due to the high antibiotic consumption in young children and thus leading to the emergence of antibiotic resistance and multi-drug resistance [25].

In the pre-PCV era, an increased rate of antibiotic non-susceptibility in pneumococcal meningitis was observed in many sites worldwide [5-8,13,14,26,27]. This increase supports the well-known phenomenon of increased antibiotic resistance following the continuous usage of antibiotic, driven by selective pressure [24]. In southern Israel, seasonal variations in antibiotic resistance among otitis media pneumococcal isolates were associated with substantial variations in antibiotic consumption [28]. Furthermore, increased resistance to multiple antibiotics among nasopharyngeal carried pneumococcal isolates was associated with increased azithromycin consumption [29]. A recent study from southern Israel showed reduction in overall antibiotics dispatched prescription rates in children following PCV7/PCV13 sequential introduction [30], further supporting our hypothesis. Following PCV7 introduction, a substantial decrease in pneumococcal meningitis caused by PCV7 serotypes was observed, along with an increase in disease caused by non-vaccine serotypes, including strains non-susceptible to antibiotics [12,18,19,31]. Similarly, following PCV13 introduction, a substantial decline in PCV13-serotypes disease rates, along with an increase in non-VT13 serotypes were observed [17,23,32-34].

The reduction in disease caused by VT13 serotypes is accompanied by a reduction in the carriage of vaccine serotypes [24] resulting in decreased ‘antibiotic pressure’. In the pre-PCV era, vaccine-serotypes were the main pneumococcal serotypes carried and responsible for causing disease; consequently, these were the main serotypes exposed to antibiotic pressure. It is not surprising, therefore, that these serotypes were the main strains exhibiting antibiotic resistance in the pre-PCV era. In contrast, non-vaccine serotypes were less exposed to antibiotic pressure and with the near elimination of vaccine-serotypes, the main burden of antibiotic resistance was eliminated. Nevertheless, it is important to recognise the possibility of new emerging antibiotic resistant strains among non-vaccine serotypes, deriving from the time elapsed since PCV13 introduction and increased exposure of the now predominating non-vaccine serotypes to antibiotic pressure. Indeed, following PCV7 introduction in the US, rapid replacement with penicillin-non-susceptible non-vaccine serotypes was observed [35]. However, when the overall impact on antibiotic resistance was evaluated, the outcome was positive.

Currently in Israel, treatment of pneumococcal meningitis in children involves empiric addition of vancomycin to ceftriaxone. It was previously suggested that in the epidemiologic setup of low ceftriaxone resistance (< 1%), it may be suitable to treat suspected pneumococcal meningitis cases empirically with ceftriaxone only, without adding vancomycin to the treatment regimen [3]. With this in mind, it may be suitable to recommend ceftriaxone only as an empiric treatment for pneumococcal meningitis in Israel (and possibly other settings where ceftriaxone resistance is low) assuming there is suitable ongoing surveillance. A possible advantage to removing vancomycin from the empiric treatment of meningitis would be in the reduction of potential side effects to the drug, including the emergence of strains resistant to vancomycin.

Continuous surveillance is needed to better understand and track antibiotic resistance rates, as well as identifying emerging resistant serotypes. In addition, surveillance could help to possibly developing new, broader (higher valency or protein based), pneumococcal vaccines.

The major strengths of our study include the utilisation of prospective, active and population-based methodology, as well as a large number of cases. The relatively long time period elapsed since PCV13 introduction to the Israeli NIP (6 years) also enables a more accurate evaluation of PCV13 impact. The main limitation of our study is the relatively high rate of undetermined serotypes early in the pre-PCV period. In recent years, however, the rate of extrapolated serotypes has dropped to < 5% of all isolated pneumococci. Notably, while in the pre-PCV period only 65% of penicillin-resistant isolates had a known serotype, this proportion decreased to 0% and has remained so since July 2009. However, while incomplete data on serotypes is a limitation, testing of penicillin resistance was done for ca 90% of isolates providing a comprehensive picture of penicillin resistance dynamics.

An additional limitation lies in the analysis of IRRs and ORs comparing grouped study years according to PCVs uptake (i.e. pre-PCV, PCV7 and PCV13 period) and the relatively small sample size in each year. Nevertheless, incidence rates for overall pneumococcal meningitis and antibiotic resistance proportions among all isolates and serotype sub-groups (VT13, non-VT13) are presented for each individual year. Additionally, chi-square for linear trend was calculated for penicillin resistance rates (proportions) and showed statistically significant reduction.

In summary, PCV7/PCV13 sequential introduction resulted in > 80% reduction of penicillin-resistant incidence rates of pneumococcal meningitis, in parallel with the disappearance of ceftriaxone resistant disease in Israeli children aged 5 years or younger. These trends should be considered by physicians when choosing an empiric treatment for pneumococcal meningitis.
